# High Seroprevalence of IgG Antibodies to Multiple Arboviruses in People Living with HIV (PLWHIV) in Madagascar

**DOI:** 10.3390/v15112258

**Published:** 2023-11-15

**Authors:** Fetra Angelot Rakotomalala, Julie Bouillin, Santatriniaina Dauphin Randriarimanana, Guillaume Thaurignac, Luca Maharavo, Mihaja Raberahona, Lucien Razafindrakoto, Jasmina Rasoanarivo, Mala Rakoto-Andrianarivelo, Danielle Aurore Doll Rakoto, François Xavier Babin, Tahinamandranto Rasamoelina, Eric Delaporte, Luc Hervé Samison, Martine Peeters, Eric Nerrienet, Ahidjo Ayouba

**Affiliations:** 1 Centre d’Infectiologie Charles Mérieux, Université d’Antananarivo, Antananarivo 101, Madagascar; fetrangelot@gmail.com (F.A.R.); rshdauphin@gmail.com (S.D.R.); lucjonah@gmail.com (L.M.); mala.rakoto@hotmail.fr (M.R.-A.); ninamandranto@yahoo.fr (T.R.); drsamison@yahoo.fr (L.H.S.); 2 TransVIHMI, University of Montpellier, Institut National de la Santé et de la Recherche Médicale (INSERM), Institut de Recherche pour le Développement (IRD), 34394 Montpellier, France; julie.bouillin@ird.fr (J.B.); guillaume.thaurignac@ird.fr (G.T.); eric.delaporte@ird.fr (E.D.); martine.peeters@ird.fr (M.P.); 3 Ecole Doctorale Sciences de la Vie et de l’Environnement, Université d’Antananarivo, Antananarivo 101, Madagascar; dad.rakoto@yahoo.fr; 4 Service des Maladies Infectieuses, Centre Hôspitalier Universitaire Joseph Raseta de Befelatanana, Antananarivo 101, Madagascar; raberahona@gmail.com; 5 Service de Pneumo-Phtisiologie, Centre Hospitalier Universitaire Analakininina, Toamasina 501, Madagascar; drrazafindrakoto@yahoo.fr; 6 Secrétariat Exécutif du Comité National de la Lutte Contre le SIDA, Antananarivo 101, Madagascar; jasminahrasoanarivo@gmail.com; 7 Fondation Mérieux, 69002 Lyon, France; fx.babin@fondation-merieux.org (F.X.B.); eric.nerrienet@fondation-merieux.org (E.N.)

**Keywords:** HIV-1, PLWHIV, arbovirus, Usutu virus, Dengue virus, Chikungunya virus, West Nile virus, O’nyong nyong virus, Zika virus, Madagascar

## Abstract

To estimate the prevalence of IgG antibodies against six arboviruses in people living with HIV-1 (PLWHIV) in Madagascar, we tested samples collected between January 2018 and June 2021. We used a Luminex-based serological assay to detect IgG antibodies against antigens from Dengue virus serotypes 1–4 (DENV1–4), Zika virus (ZIKV), West Nile virus (WNV), Usutu virus (USUV), Chikungunya virus (CHIKV), and O’nyong nyong virus (ONNV). Of the 1036 samples tested, IgG antibody prevalence was highest for ONNV (28.4%), CHIKV (26.7%), WNV-NS1 (27.1%), DENV1 (12.4%), USUV (9.9%), and DENV3 (8.9%). ZIKV (4.9%), DENV2 (4.6%), WNV-D3 (5.1%), and DENV4 (1.4%) were lower. These rates varied by province of origin, with the highest rates observed in Toamasina, on the eastern coast (50.5% and 56.8%, for CHIKV and ONNV, respectively). The seroprevalence increased with age for DENV1 and 3 (*p* = 0.006 and 0.038, respectively) and WNV DIII (*p* = 0.041). The prevalence of IgG antibodies against any given arborvirus varied over the year and significantly correlated with rainfalls in the different areas (r = 0.61, *p* = 0.036). Finally, we found a significant correlation between the seroprevalence of antibodies against CHIKV and ONNV and the HIV-1 RNA plasma viral load. Thus, PLWHIV in Madagascar are highly exposed to various arboviruses. Further studies are needed to explain some of our findings.

## 1. Introduction

Arboviruses represent a large and diverse family of more than five hundred species of viruses distributed initially in the intertropical regions of Africa, Asia, and the Americas [1,2]. However, multiple factors, including climate and land use changes, deforestation, uncontrolled urbanization, international trade, and the failure of vector control programs, have led to the extension of the area of presence of arboviruses beyond the tropics [3,4,5,6,7,8,9,10]. Of the more than 500 arboviral species registered so far, it is assumed that 100–150 are pathogenic to humans and animals [11]. Among these pathogenic arboviruses, some, like Dengue virus, are very ancient and affect annually an estimate of 390 million individuals worldwide [12]. The increasing frequency and magnitude of outbreaks of arbovirus-caused diseases such as yellow fever, Zika, Dengue, Chikungunya, and others led the World Health Organization (WHO) to launch in March 2022 the Global Arbovirus Initiative [13]. This strategic plan’s main objective is to tackle emerging and re-emerging arboviruses with epidemic and pandemic potential, with a special focus on risk monitoring, pandemic prevention, preparedness, detection, and response.

Multiple present-day arboviruses likely originated in Africa. Yellow fever virus (YFV) is known to have circulated endemically in Sub-Saharan Africa for centuries [14]; West Nile virus (WNV) and Zika virus (ZIKV) were first identified in Uganda in 1937 and 1947, respectively [15]; Chikungunya virus (CHIKV) was identified in 1952 in Tanzania [16]; and Usutu virus (USUV) was identified in 1959 in South Africa. In 2015, YFV, one of the rare vaccine-preventable arboviral diseases, re-emerged in Angola and spread to the neighboring country of the Democratic Republic of Congo and beyond, to Kenya and China, causing thousands of cases and hundreds of fatalities [17,18]. Like in many other African countries, arthropod-borne diseases are a public health threat in Madagascar. Entomological and epidemiological surveys showed evidence of the circulation of arboviruses in domestic and wild animals as well as in humans [19,20,21,22]. For example, serological surveys by teams from the Institut Pasteur of Madagascar showed evidence of antibodies against Chikungunya, Dengue, Zika, yellow fever, West Nile, and Rift Valley fever viruses in Nosy Be [23]. In 2011, a 58-year-old woman returning from Madagascar died in La Réunion as a result of neuroinvasive West Nile virus disease [24]. Finally, Madagascar experienced several arbovirus outbreaks, including RVFV, in 1990 [25,26], and Dengue and Chikungunya outbreaks in 2006 [27]. Finally, a very recent report [28] on 1680 samples collected between 2011 and 2013 in the general population tested for IgG antibodies against Dengue, Chikungunya, and West Nile viruses. The authors observed an overall seroprevalence ranging between 6.5% and 13.7% for Dengue, Chikungunya, and West Nile viruses [28].

Thus, several arboviruses are endemic or emerging in Madagascar, and people living therein are exposed to these viruses. Type 1 human immunodeficiency virus (HIV-1) is another infectious disease representing a public health threat to Malagasy. According to the latest UNAIDS report, there is an estimate of 70,000 people living with HIV (PLWHIV) in Madagascar [29]. Seroprevalence in the general population is low but high in key populations (sex workers, men who have sex with men, etc.) [29]. Data on HIV from Madagascar are scarce, and those available on the situation of HIV-1-infected persons are old and/or limited in sample size. Additional data, like co-infections and the immune status of this vulnerable population, are even more scarce. Our objective in this work is to better characterize this specific population by describing the co-infections that affect them and, in ongoing work, by describing their HIV-positive status, beyond viral load, by determining HIV-1 genetic diversity and drug-resistance mutations for better care. Because HIV-1 infection is a vulnerability, additional exposure to other pathogens such as arboviruses might further impact their health.

In the present work, we investigated the seroprevalence of IgG antibodies to six arboviruses of public health importance in PLWHIV from five of the six provinces of Madagascar. Our data showed an overall high seroprevalence of antibodies against Chikungunya and O’nyong nyong, Dengue viruses 1 and 3, and West Nile virus in Madagascar.

## 2. Materials and Methods

### 2.1. Samples

**Figure 1:**
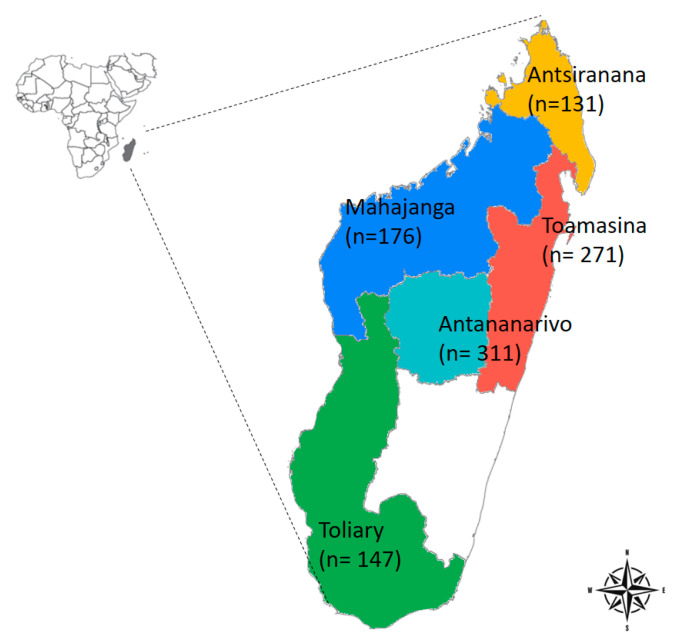
Location of sample collection sites. The figure shows the map of Madagascar, with Africa in perspective. Samples were collected in five of the six provinces of Madagascar. The numbers in parentheses represent the sample size from each of the five provinces.

Plasma samples were collected from PLWHIV in Madagascar in the framework of their virological follow up. The patients originated from five of the six provinces of Madagascar and were enrolled between March 2018 and June 2021. The number of patients included and the province they originated from are depicted in Figure 1. The plasma samples were stored at −80 °C in the biobank of the Centre d’Infectiologie Charles Mérieux (CICM) in Antananarivo, the capital city, until their use.

### 2.2. Detection of IgG Antibodies to Arboviruses

To detect IgG antibodies against six species of arboviruses, we used a multiplex assay based on the Luminex technology as described earlier [30]. The assay includes antibodies against the recombinant E2 protein of Chikungunya and O’nyong-nyong and the NS1 protein of Dengue serotypes 1–4, Usutu, West Nile, and Zika viruses. In addition, the envelope domain-3 protein of West Nile virus was included. Briefly, we covalently coupled recombinant proteins to carboxyl-functionalized fluorescent magnetic beads (Luminex Corp., Austin, TX, USA) with the BioPlex amine-coupling kit (Bio-Rad Laboratories, Marnes-la-Coquette, France) according to the manufacturer’s instructions. We blocked unreacted sites with blocking buffer from the amine-coupling kit. We washed the protein-coupled microsphere with PBS and stored it in storage buffer (Bio-Rad, Hercules, CA, USA) at 4 °C in the dark until use. Serum or plasma samples diluted 1/200 in sample buffer were used and processed as extensively described earlier [30]. Binding results are expressed as median fluorescence intensity (MFI) per 50 beads. At the end of the run on the BioPlex200 platform, data were exported into a Microsoft Excel 2016 spreadsheet for further processing. Overall, the assay detects antibodies against 10 different antigens simultaneously. When evaluated on a panel of human samples with known arboviral status, each of the 10 antigens of the assay presented a sensitivity and a specificity equal or superior to 95% [30].

### 2.3. Plasma HIV-1 RNA Quantification

HIV-1 viral RNA was extracted from 250 µL of plasma using an automated nucleic acid extractor with a GXT NA extraction kit (Bruker Life Science GenoXtract^®®^, Biocentric, Bandol, France) as per the manufacturer’s instructions. A volume of 10 µL of extracted RNA was used for HIV-1 RNA quantification with the Generic HIV viral load kit (Biocentric, Bandol, France) as described earlier [31]. The threshold of quantification with this assay was 250 RNA copies/mL of plasma.

### 2.4. Statistical Analyses

To compare two categories, we used the nonparametric Mann–Whitney U test. To compare three or more categories, the Kruskal–Wallis test was used. To evaluate the evolution of seroprevalence over time, we used the chi-square test for trends. Finally, we used person correlation to evaluate the strength of the relationship between arbovirus seroprevalence and rainfall in Madagascar. For all the statistical tests, the threshold of significance was set to 0.05.

## 3. Results

### 3.1. Prevalence of IgG Antibodies against Arboviruses in PLWHIV of Madagascar

**Figure 2:**
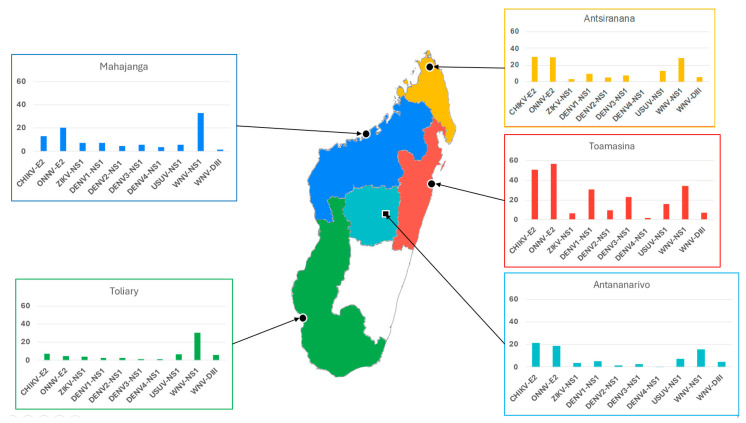
Seroprevalence of IgG antibodies to multiple arboviruses in people living with HIV-1 from Madagascar. The figure represents graphically the seroprevalence of IgG antibodies to the ten arboviral antigens studied in the five provinces of Madagascar. The data are also detailed in Table 1.

The study included randomly selected 1036 PLWHIV followed up with at the CICM. They originated from five provinces of Madagascar, with a sex ratio of 1. The overall IgG antibody seroprevalence varied between 28.4% for ONNV E2 antigen to 1.4% for DV4 NS1 antigen (Table 1). The seroprevalence of IgG antibodies to anti-CHIKV E2 antigen (26.7%) was comparable to that of ONNV E2, the other alphavirus studied. In the flavivirus family, whose antigens were included in our assay, the seroprevalence of IgG antibodies to WNV NS1 was the highest, at 27.1%, followed by DENV serotype 1, at 12.4%, and USUV NS1, at almost 10%. Antibodies to other DENV serotypes, ZIKV NS1 and WNV DIII antigens, although detectable, were all below 10%. The stratification of samples by province of origin showed that, for all of the antigens tested, the highest seroprevalence was observed in the Toamasina province. For example, 56.83%, 50.55%, 34.32%, 30.63%, and 22.88% of the samples tested positive for antibodies against ONNV E2, CHIKV E2, WNV NS1, DV1 NS1, and DV3 NS1, respectively. Inversely, the seroprevalence was lowest in the Toliary province, on the western coast of Madagascar, for all of the tested antigens except for WNV NS1, for which the seroprevalence was 30.61% (Table 1 and Figure 2).

### 3.2. IgG Seroprevalence to Multiple Arboviruses in Madagascar Stratified by Sex, Age, and Sampling Period

We analyzed our data by stratifying the IgG seroprevalence by the sex of the patients (Table 2). For all 10 antigens tested, the proportion of reactive samples in both groups was not statistically different, with the exception of USUV NS1, for which there were more reactive samples in males than in females (*p* = 0.003). When we analyzed our data by stratifying by age categories (i.e., children, infants, adolescents, adults, and those over 55 years old), we observed statistically significant differences for DENV1 (*p* = 0.006), DENV3 (*p* = 0.038), and WNV DIII (*p* = 0.041) antigens (Table 3). For WNV NS1, there was a trend with a *p*-value of 0.143. Of note, for all of the antigens tested, there was a tendency towards an increase in the proportion of positive samples with age. This was especially visible for all of the flavivirus antigens, for which the proportion of positive samples in the age category of 0–5 years old was zero (Table 3).

Arboviruses are insect-vectored pathogens that are season and climate sensitive. We thus analyzed our data after stratification by year and month of sampling. We observed statistically significant seroprevalence variation over the four years covering the study period for 7 of the 10 tested antigens, namely, CHIKV E2, ONNV E2, DENV1 NS1, DENV2 NS1, DENV3 NS1, USUV NS1, and WNV DIII (Table 4a). For ZIKV NS1, DENV4 NS1, and WNV NS1, the variation trend was not statistically significant during this study period. We further split the study period by month of sampling (Table 4b). Despite the limited sample size for some months (e.g., April, n = 28; May, n = 33; and July, n = 26), the significant prevalence variation trend observed on a yearly basis was maintained by month for six of the seven antigens mentioned above. The only arbovirus antigen whose monthly prevalence variation became statistically non-significant was that of WNV DIII.

### 3.3. Correlation of IgG Seroprevalence and Rainfall in Madagascar

**Figure 3:**
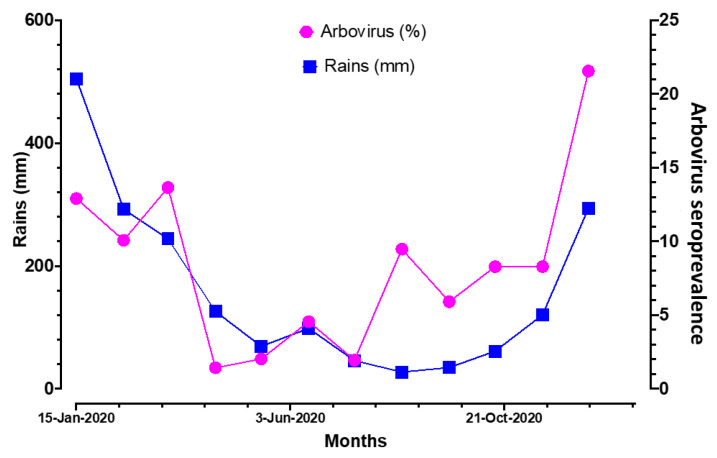
Variation in seroprevalence of antibodies against arboviruses in Madagascar over time. The left axis (blue squares) represents the average rainfall (in millimeters), and the right axis (pink circles) represents the seroprevalence of IgG antibodies against arboviruses (in percentage). The horizontal axis represents the months of sampling averaged over the four years of the study period. A significant correlation was observed between rainfall and seroprevalence (Pearson, r = 0.61, *p* = 0.036).

One of the correlates of insect-borne pathogen transmission in tropical or subtropical regions is the season (rainy vs. dry season). To assess whether the variations observed in Table 4b were linked to rainfall, we collected pluviometry data registered in Madagascar during the four years of the study period. We plotted the averaged four-year rain data together with the global arbovirus seroprevalence observed in our study. The results from this analysis (Figure 3) showed a high and significant correlation (r = 0.61, *p* = 0.036) between rainfall and arbovirus seroprevalence. In general, IgG antibody levels to any given arbovirus antigen increased with rainfall intensity and vice versa.

### 3.4. Impact of HIV-1 Plasma Viral Load on IgG seroprevalence to Arboviruses

The study population of the present work consisted of people living with HIV followed up with for this infection. The majority of them, 70% (726/1036), were on highly active antiretroviral therapy (HAART) consisting of a combination of efavirenz, lamivudine, and tenofovir. The remaining 30% (310/1036) were antiretroviral naïve at the time of sampling. Patients presenting an HIV-1 plasma viral load below 1000 RNA copies/mL are considered to be controlling the infection, whereas those presenting values above that threshold are considered to be in virological failure. We stratified our study population into these two categories and compared the seroprevalence to each of the 10 antigens tested (Table 5). We found that only the two alphaviruses (Chikungunya and O’nyong nyong) presented statistically significant differences between the two HIV-1 plasma viral load categories.

## 4. Discussion

Our objective in this work was to evaluate the prevalence of IgG antibodies to some arboviruses of public health importance in people living with HIV from Madagascar. To that end, we used a multiplex assay previously validated on human and animal samples [30,32].

We found high seroprevalence of IgG antibodies to the two alphaviruses of our panel (CHIKV and ONNV), WNV, DENV 1, and, to a lower level, USUV and DENV-3. (Table 1). CHIKV and ONNV are the two most important members of the alphavirus genus (which accounts for up to 40) and are associated with disease in humans [33,34]. However, cases of ONNV infections are very likely misdiagnosed or underreported because of symptoms similarities with CHIKV cases and the lack of specific assays. In the present work, we observed comparable (high) prevalence of IgG antibodies against CHIKV and ONNV, indicative of co-circulation of both viruses or cross-reactions between the two E antigens of the two viruses or other related viruses not included in the study. In our earlier work, we observed 40–60% cross-reactions between CHIKV and ONNV E2 antigens [32]. Further studies, including seroneutralization and molecular characterization of viruses from acute patients, should be performed to confirm the presence of ONNV in Madagascar. Nevertheless, the seroprevalence we observed for the different arboviruses is in line with that from earlier works in the late 1970s and 1980s by teams from the Institut Pasteur of Madagascar [23,35,36,37,38]. For example, in a work performed in 1977, 27 of 56 samples tested (48.2%) by agglutination assay presented a titer of at least 1/10 against 1 of 16 arboviruses screened, including Chikungunya, yellow fever, Dengue 1 and 3, West Nile, and Zika viruses. Of note, only 10/56 samples presented agglutination titers of 1/40 and 12/56. In the same report, the authors also tested 215 samples collected in 1986 and found that 101/215 (46.9%) were reactive to at least one arbovirus antigen with a titer of 1/10 or more [23]. Hence, despite the weak sensitivity and specificity of hemagglutination assay compared to modern tools, several arboviruses studied here appear to have been circulating in Madagascar for years. Our findings are also in line with the recent outbreaks of West Nile, Dengue, and Chikungunya in the Indian Ocean’s islands, including Madagascar [24,25,27]. A recent study reported on the seroprevalence of IgG antibodies to Dengue, Chikungunya, and West Nile viruses in Madagascar [28]. The authors found an overall seroprevalence of 6.5%, 13.7%, and 12.7% of IgG antibodies to Dengue, Chikungunya, and West Nile viruses, respectively. The seroprevalence varied significantly by the region of sampling, reaching 86% for Chikungunya in Nosy Be, for example. We also observed high seroprevalence of IgG antibodies against alphaviruses in our study (up to 56% in Toamasina, Table 1) despite differences in diagnostic tools used, the sampling period (2011–2013 versus 2018–2021), and the study population (general population vs. PLWHIV). Nevertheless, the two reports underscore an endemic circulation of arboviruses in Madagascar.

Surprisingly, a significant proportion of samples (9.9%) presented IgG antibodies to the Usutu virus antigen. Usutu virus was first identified in South Africa in 1959, is mainly transmitted by birds and possibly bats, and represents an emerging threat in multiple parts of the world [39]. In Africa, data on Usutu virus remain scarce, and our present report is among a handful of studies on this emerging pathogen [40,41,42]. Investigations on Usutu virus and its potential impacts should thus be implemented in Madagascar not only because of data scarcity but also in view of the findings presented in Table 2, where we stratified the data by sex. Indeed, the only antigen for which a statistically significant difference was observed was the Usutu antigen. We are unable to explain this finding based on our current knowledge of the Usutu virus life cycle in Madagascar. In a work cited above [23], the authors found no link between seroprevalence and sex in the arboviruses they included in their screening, but the assay then used did not contain Usutu virus antigens.

The severity of most infectious diseases is age related because pathogen clearance from the body relies, in part, on the immune competence of the host. Since our study population comprised an extended range of ages, we stratified it into categories (Table 3). We observed a statistically significant trend towards an increase in seroprevalence with age for antibodies against DENV-1, DENV-3, and WNV-DIII antigens. Interestingly, we detected no IgG antibodies against any of the flavivirus antigens tested in the age category of less than 5 years old, whereas this was not the case for the alphaviruses Chikungunya and O’nyong-nyong, although the number of samples was limited. The fact that seroprevalence increased with age underscores the continuous exposure of the human population to the pathogens studied and, hence, their endemicity. A similar observation was made for WNV in Madagascar [22,43], for DENV and Rift Valley fever (RVF) in Kenya [44], and for DENV and CHIKV in Ecuador [45].

Our data also revealed the variation in seroprevalence with time and its correlation with local rainfall. This is consistent and not a surprising observation for vector-borne diseases like arboviruses. Despite the fact that IgG antibodies are not indicative of acute infection, our observations indirectly reflect arbovirus vector dynamics in Madagascar, as extensively described by entomological investigations [38,46,47].

Finally, since our study population was people living with HIV, we analyzed our data by stratifying only by HIV viral load status, because the latest WHO guidelines for HIV patient care recommend the use of viral load quantification instead of CD4 cell enumeration. Patients failing treatment are known to be more at risk of opportunistic infections. Two studies were performed on HIV-positive cohorts in Martinique and Guadeloupe islands after the 2014 CHIKV outbreak. In one study, the authors found no difference in seroprevalence of antibodies against CHIKV when they stratified the patients by HIV plasma viral load (<50 copies/mL vs. ≥50 copies/mL). However, this threshold is more stringent than the one we used in our study (1000 copies/mL), which might be enough to explain the difference in our observations [48]. The second study analyzed CD4- and CD8-positive T cells in HIV-positive patients with confirmed CHIKV infection. They found a significant drop in both cell types during the acute phase of CHIKV, with a return to the pre-acute phase of CHIKV infection a few weeks later [49]. CHIKV infection, like many other viral infections, induces a cytokine storm during the acute phase, with the release of important amounts of cytokines, including pro-inflammatory cytokines, which are also known to stimulate HIV replication in T cells. A large meta-analysis showed that up to 10 pro-inflammatory and 4 anti-inflammatory cytokines were found to be increased in the acute phase of CHIKV infection [50]. From these observations and our own findings, it thus remains difficult to explain the link between the prevalence of the two alphaviruses and HIV plasma viral load because the detected antibodies are IgGs, and they appear after the acute phase of CHIKV infection. Further investigations are thus needed—for example, a study of the clinical and immunological impact of arbovirus infection in the general population in Madagascar.

### Limitations of the Study

The present work has some limitations. The first limitation is the study population, which consisted of HIV-positive patients. They are not representative of the general Malagasy population, and the results might be biased by this fact. However, PLWHIV in Madagascar are living in the same environment as people not infected by HIV and subjected to the same life peculiarities, and the recent report by Broban and co-workers [28] showed that, for the three pathogens common to both studies, the trends are similar. The second limitation of our work is the absence of additional, discriminatory tests, like seroneutralization, specifically for alphaviruses, to differentiate CHIKV and ONNV infections.

## 5. Conclusions

We showed in the present work that PLWHIV in Madagascar are highly exposed at variable levels to multiple arboviruses regardless of their province of origin. This exposure is season sensitive. We showed evidence, for the first time, of circulation of Usutu virus in humans in Madagascar. Appropriate vector control measures should be urgently undertaken to mitigate arbovirus circulation in Madagascar. Some of the findings reported here, like the link between the seroprevalence of IgG antibodies and the sex of the patients or the seroprevalence of IgG antibodies against alphaviruses and HIV-1 plasma viral load, need further investigation.

## Figures and Tables

**Table 1:** Seroprevalence of IgG antibodies to multiple arboviruses in people living with HIV-1 (PLWHIV) from Madagascar.

Provinces	CHIKV * E2	ONNV E2	ZIKV NS1	DENV1 NS1	DENV2 NS1	DENV3 NS1	DENV4 NS1	USUV NS1	WNV NS1	WNV DIII
Antananarivo (n = 311)	21.54	18.97	3.54	5.14	1.61	2.57	0.32	7.40	15.43	4.82
Antsiranana (n = 131)	29.77	29.01	3.05	9.16	4.58	7.63	0.00	12.98	28.24	5.34
Mahajanga (n = 176)	13.07	20.45	7.39	7.39	4.55	5.68	3.41	5.68	32.95	1.70
Toamasina (n = 271)	50.55	56.83	6.27	30.63	9.23	22.88	1.85	15.87	34.32	7.01
Toliary (n = 147)	7.48	4.76	4.08	2.72	2.72	1.36	1.36	6.80	30.61	6.12
Overall prevalence (n = 1036) 95% CI	26.7 (24.0–29.4)	28.4 (26.6–31.1)	4.9 (3.6–6.2)	12.4 (10.4–14.4)	4.6 (3.4–5.9)	8.9 (7.1–10.6)	1.4 (0.6–2.1)	9.9 (8.1–11.8)	27.1 (24.4–29.8)	5.1 (3.8–6.5)

* CHIKV-E2: Chikungunya virus E2 protein; ONNV-E2: O’nyong n’yong virus E2 protein; ZikV-NS1: Zika virus NS1 protein; DENV1–4-NS1: Dengue virus serotypes 1–4 NS1 proteins; USUV-NS1: Usutu virus NS1 protein; WNV-NS1: West Nile virus NS1 protein; WNV-DIII: West Nile virus domain 3 protein.

**Table 2:** Seroprevalence of IgG antibodies to multiple arboviruses in people living with HIV-1 (PLWHIV) from Madagascar, stratified by sex.

Sex	CHIKV E2	ONNV E2	ZIKV NS1	DENV1 NS1	DENV2 NS1	DENV3 NS1	DENV4 NS1	USUV ** NS1	WNV NS1	WNV DIII
F (n = 518)	28.76	28.19	5.21	12.16	4.25	8.30	1.35	7.14	29.15	5.02
M (n = 518)	24.71	28.57	4.63	12.55	5.02	9.46	1.35	12.74	25.10	5.21

** *p* = 0.003.

**Table 3:** Seroprevalence of IgG antibodies to multiple arboviruses in people living with HIV-1 (PLWHIV) from Madagascar, stratified by age.

Age Group (Years)	CHIKV E2	ONN E2	ZIKV NS1	DENV1 NS1	DENVV2 NS1	DENVV3 NS1	DENVV4 NS1	Usutu NS1	WNV NS1
(0–5); n = 10	20.00	10.00	0.00	0.00	0.00	0.00	0.00	0.00	0.00
(5–15); n = 11	36.36	27.27	9.09	9.09	9.09	18.18	0.00	0.00	9.09
(15–25); n = 174	24.71	25.86	4.02	5.17	1.72	4.60	0.00	10.34	26.44
(25–55); n = 794	27.58	29.22	5.04	13.60	5.16	9.32	1.64	9.82	28.21
>55; n = 47	19.15	27.66	6.38	21.28	6.38	17.02	2.13	14.89	21.28
*p*-*value (X*^2^*)*	0.594	0.645	0.833	**0.006**	0.273	**0.038**	0.498	0.456	0.143

**Table 4:** (a) Seroprevalence of IgG antibodies to multiple arboviruses in people living with HIV-1 (PLWHIV) from Madagascar, stratified by year of sampling. (b) Seroprevalence of IgG antibodies to multiple arboviruses in people living with HIV-1 (PLWHIV) from Madagascar, stratified by month of sampling.

**Year of Sampling**	CHIKV * **E2**	**ONNV** **E2**	**ZIKV** **NS1**	**DENV1** **NS1**	**DENV2** **NS1**	**DENV3** **NS1**	**DENV4** **NS1**	**USUV** **NS1**	**WNV** **NS1**	**WNV** **DIII**
2018 (n = 23)	8.70	34.78	8.70	8.70	4.35	0.00	0.00	4.35	39.13	8.70
2019 (n = 322)	28.57	29.50	4.97	9.63	3.42	7.76	0.31	9.32	26.40	2.48
2020 (n = 502)	30.08	31.47	5.78	16.33	6.57	11.95	1.99	12.95	28.09	6.77
2021 (189)	16.93	17.46	2.12	6.88	1.59	3.70	1.59	3.70	24.34	4.76
***p (X* ^2^ *for trend):***	**0.001**	**0.003**	0.198	**0.002**	**0.025**	**0.002**	0.207	**0.003**	0.433	**0.044**
**Month of Sampling**	**CHIKV** **E2**	**ONNV** **E2**	**ZIKV** **NS1**	**DENV1** **NS1**	**DENV2** **NS1**	**DENV3** **NS1**	**DENV4** **NS1**	**USUV** **NS1**	**WNV** **NS1**	**WNV** **DIII**
January (n = 88)	42.05	44.32	5.68	21.59	10.23	19.32	2.27	15.91	30.68	4.55
February (n = 70)	31.43	35.71	7.14	31.43	12.86	22.86	1.43	14.29	28.57	7.14
March (n = 110)	33.64	38.18	5.45	18.18	4.55	10.00	0.00	15.45	33.64	7.27
April (n = 28)	21.43	14.29	0.00	3.57	0.00	3.57	3.57	0.00	17.86	3.57
May (n = 33)	21.21	21.21	0.00	6.06	0.00	0.00	0.00	3.03	24.24	6.06
June (n = 92)	7.61	11.96	3.26	5.43	1.09	3.26	1.09	5.43	20.65	6.52
July (n = 26)	30.77	19.23	3.85	0.00	0.00	0.00	0.00	19.23	23.08	3.85
August (n = 94)	28.72	28.72	4.26	14.89	6.38	8.51	2.13	12.77	26.60	2.13
September (n = 86)	13.95	16.28	4.65	6.98	3.49	3.49	3.49	8.14	25.58	5.81
October (n = 93)	29.03	26.88	5.38	6.45	3.23	3.23	1.08	9.68	25.81	8.60
November (n = 108)	23.15	2.99	0.58	0.58	0.19	0.58	0.10	0.29	2.80	0.19
December (n = 208)	29.81	30.77	5.77	12.98	4.81	11.54	0.96	9.62	28.37	4.33
*p (X* ^2^ *for trend):*	**<10^−4^**	**<10^−4^**	0.919	**<10^−4^**	**0.005**	**<10^−4^**	0.734	**0.008**	0.809	0.608

**Table 5:** Seroprevalence of IgG antibodies to multiple arboviruses in people living with HIV-1 (PLWHIV) from Madagascar, stratified by HIV plasma viral load detectability.

HIV Viral Load	CHIKV * E2	ONNV ** E2	ZIKV NS1	DENV1 NS1	DENV2 NS1	DENV3 NS1	DENV4 NS1	USUV NS1	WNV NS1	WNV DIII
VL < 1000 cp/mL (n = 580)	31.52	32.43	6.35	17.69	7.26	12.70	2.04	13.38	33.56	6.58
VL ≥ 1000 cp/mL (n = 450)	43.31	47.45	7.32	15.61	5.10	11.46	1.59	14.01	41.40	7.64

* *p* = 0.024; ** *p* = 0.003.

## Data Availability

The raw Luminex data are available to the scientific community upon a simple request to the corresponding author.
